# P-1825. Investigating the Impact of Immunomodulatory Drugs on Severe COVID-19 Through Transcriptomic Analysis

**DOI:** 10.1093/ofid/ofae631.1988

**Published:** 2025-01-29

**Authors:** Jeong Yeon Kim, Yoo Jin Sung, Eujin Hong, Hyun Uk Kim, Young Kyung Yoon

**Affiliations:** Korea University College of Medicine, Seoul, Seoul-t'ukpyolsi, Republic of Korea; Korea Advanced Institute of Science and Technology, Daejeon, Taejon-jikhalsi, Republic of Korea; Korea Advanced Institute of Science and Technology, Daejeon, Taejon-jikhalsi, Republic of Korea; Korea Advanced Institute of Science and Technology, Daejeon, Taejon-jikhalsi, Republic of Korea; Korea University College of Medicine, Seoul, Seoul-t'ukpyolsi, Republic of Korea

## Abstract

**Background:**

The Coronavirus disease (COVID-19) has highlighted the role of hyperinflammatory states and cytokine storms in severe SARS-CoV-2 infections, leading to extensive research into immunomodulatory drugs previously used for autoimmune diseases. Despite their promising effects, the mechanisms underlying their efficacy remain unclear. This study aims to elucidate these mechanisms through RNA-sequence data analysis.

**Table 1.**
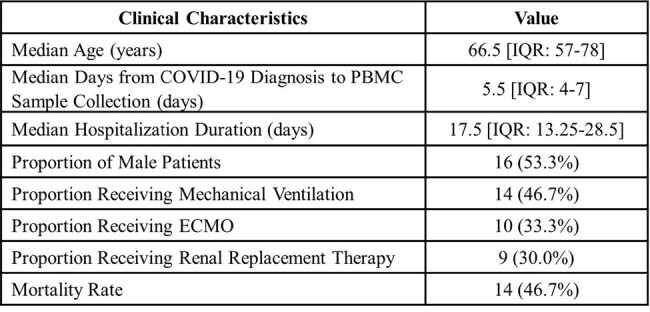
Clinical characteristics of 30 COVID-19 patient cohort

**Methods:**

Bulk RNA sequencing data from PBMC samples of COVID-19 patients was used to identify two sets of differentially expressed genes (DEGs). The first set was made by comparing COVID-19 patient data to healthy individuals using public datasets, while the second set involved DEGs from samples treated with immunomodulatory drugs versus untreated samples. Comparative analysis focused on genes with opposite expression changes, particularly those showing contrasting fold changes between COVID-19 and drug-treated samples.

**Figure 1.**
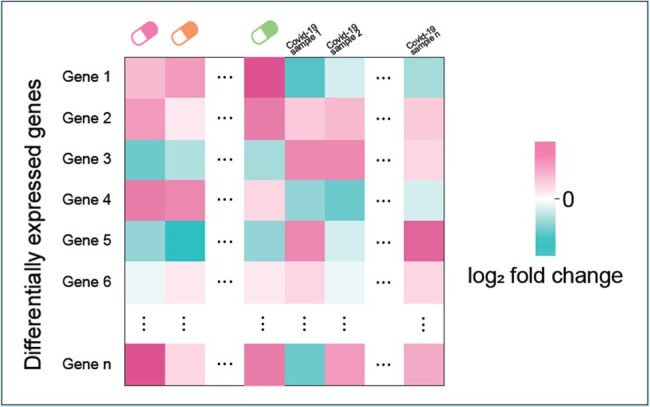
DEG array

**Results:**

All COVID-19 patients met the criteria for severe or critical illness, requiring at least a high-flow nasal cannula. The median age was 66.5 years. Clinically, 46.7% underwent mechanical ventilation and the mortality rate stood at 46.7%. By arraying the table with all the samples combined from COVID-19 patient samples and immunomodulatory drug samples, DEGs that show opposite fold change signs were selected as having potential significance in COVID-19 pathways and being affected by immunomodulatory drugs. Specifically, 18 genes were upregulated in response to COVID-19 yet downregulated in immunomodulatory drug use, including key inflammatory and immune response genes such as FAP, RGS16, and IDO1. Conversely, 39 genes showed downregulation in response to COVID-19 but were upregulated in immunomodulatory drug use, featuring genes like DUSP1, TOB1, and RGMA.

**Table 2.**
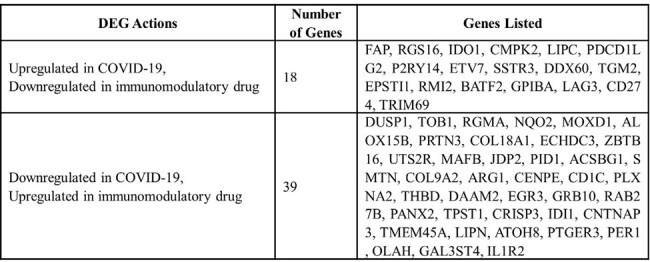
List of genes found in DEG array

**Conclusion:**

Through transcriptomic analysis and differential expression screening between COVID-19 and medication-treated samples, 57 were found to exhibit reversed fold changes in expression. Further investigation of these genes is expected. This research aims to uncover the shared biological mechanisms between autoimmune diseases and COVID-19, which could lead to effective drug repurposing strategies for treating COVID-19.

**Disclosures:**

**All Authors**: No reported disclosures

